# Mandarin functional MRI Language paradigms

**DOI:** 10.1002/brb3.525

**Published:** 2016-08-03

**Authors:** He Ci, Andre van Graan, Gloria Gonzálvez, Pamela Thompson, Andrea Hill, John S. Duncan

**Affiliations:** ^1^ Department of ImagingChengdu Military Area General HospitalChengduChina; ^2^ MRI UnitChalfont Centre for EpilepsyBucksUK; ^3^ Department of Clinical and Experimental EpilepsyUCL Institute of NeurologyLondonUK

**Keywords:** bold fMRI, brain function, Chinese, language stimulus pattern, lateralization index

## Abstract

**Objective:**

The objective of this study was to implement convenient, fast, and accurate Mandarin task paradigms for functional MRI, and to locate the Chinese language functional areas in frontal and temporal lobes.

**Materials and Methods:**

Nineteen healthy Chinese volunteers participated in this study, which utilized a block design with four language tasks: auditory naming (AN), picture naming (PN), verbal fluency‐character (VFC), and verbal fluency‐letter (VFL). All functional images were preprocessed by SPM 8, followed by first‐ and second‐level analyses and lateralization index calculation.

**Results:**

Group analyses showed that for AN and PN, maximal responses were located in the right superior temporal gyrus. The picture naming‐scrambled pictures and faces contrast gave maximal responses in the left fusiform gyrus; VFC in the left middle frontal gyrus and the left superior frontal gyrus. For VFL the maximal response was in the left superior temporal gyrus. There was some inconsistency of activations for individual subjects. At a threshold of *Z* > 2.5, 10 voxels extent, activations were seen in >50% subjects for AN in the right superior temporal gyrus, the right middle frontal gyrus, and the left middle temporal gyrus, for PN in the right superior temporal gyrus and for picture naming‐scrambled pictures and faces in left inferior frontal gyrus. As a group, the lateralization index of all contrasts were left hemisphere dominant in the frontal lobes. In the temporal lobe, the hemispheric dominance differed for different contrasts.

**Conclusion:**

These Chinese language stimulus paradigms activated language areas, and the functional regions of brain in different language tasks, and can now be piloted in clinical studies.

## 1 Introduction

Epilepsy is one of the most common diseases of central nervous system, and 30% become medically intractable with temporal lobe epilepsy being a common form (Linehan, Tellez‐Zenteno, Burneo, & Berg, 2011). Anterior temporal lobe resection (ATLR) is an effective surgical treatment for refractory temporal lobe epilepsy (Hamberger, McClelland, McKhann, Williams, & Goodman, 2007), but the anterior temporal lobe plays a very important role in language, and ATLR carries a risk of postoperative language decline (Rosazza et al., 2013). It is necessary to locate and to lateralize language areas in temporal lobes before surgery, so the risks are fully understood.

Chinese is one of the most commonly used languages in the world, and nearly 1.5 billion people, about 1/6 of the world population, use Chinese as their mother tongue. Chinese characters are different from western alphabet symbols and form an ideographic syllabic logographic language. The Chinese language is also a tone language, with various dialects (Liu, 2013). In ISO 639‐3 international language code (2007), ISO classifies Chinese into Mandarin, Cantonese, Fujian, Jin, Gan and 13 other dialects. The relationship between the Chinese spoken and written language is rather complex. Chinese characters are morphemes independent of phonetic change, and the semantics are basically the same for a single Chinese character, but the tones for different dialects are mutually unintelligible, as a consequence fMRI experimental procedures for the Chinese language are relatively complex (Feng Yan‐yun, 2011).

It is important, therefore, to focus on semantics, instead of phonetics when developing an fMRI method to evaluate Chinese language function. This study used Chinese language stimuli, modified from English stimuli, in healthy volunteers, to explore activation patterns in Chinese people, and to explore the potential use in patients considering temporal lobe surgery.

## 2 Materials and Methods

### 2.1 Participants

Nineteen healthy Chinese volunteers were recruited who were native Mandarin speakers. Exclusion criteria were any neurological or psychiatric disorder, or hearing impairment. All subjects gave written consent, and the Research Ethics Committee of University College London Hospitals approved the study.

Demographic data recorded included age, gender, educational level, etc. Handedness was assessed using the Edinburgh Handedness Questionnaire (Oldfield, 1971).

### 2.2 MR data acquisition

All data were acquired using a 3.0T MR scanner (Discovery 750; GE Healthcare, Milwaukee, WI) with a 32‐channel phased array head coil and body radio frequency coil. BOLD signal acquired by single shot echo‐planar imaging (EPI), TR = 2500 ms, TE = 22 ms, Flip angle = 90°, N.E.X = 1.00, FOV=24 cm × 24 cm, Thickness = 2.4 mm, Band width = 2.5 kHz, 64 × 64 matrix, covering the whole brain, 50 slices in total. T1WI axial images using SE sequence, TR = 8.1 ms, TE = 3.2 ms, FOV = 24 cm × 24 cm, N.E.X = 1, Flip angle = 12°, Thickness = 1.2 mm, Band width = 31.25 kHz. The field of view was positioned to maximize coverage of the frontal and temporal lobes.

### 2.3 Experimental design

The experiment was a block design, alternating between the stimulation (Task) and resting (Rest) conditions. All subjects completed four tasks and had a prior explanation with a PowerPoint demonstration to ensure that they fully understood what was required.

#### 2.3.1 Auditory naming

Auditory Naming (AN) consists of 5 blocks, each block contains a 30 s stimulus task and 2 control tasks, auditory reversed (AR) and “+”, each one lasting 15 s. In the stimulus tasks, subjects were asked to name objects after hearing descriptions. For example, for “a lava filled mountain” the answer should be “Volcano”. In the control tasks, subjects were asked to count “1, 2” while hearing the auditory description in reverse which made no sense, and to rest when “+” appeared on the screen.

#### 2.3.2 Picture naming

Picture Naming (PN) consists of five blocks, each block comprising four modules: 30 s of pictures (http://crl.ucsd.edu/experiments/ipnp/1stimuli.html), of common objects, animals or plants presented as black and white line drawings; and 15 s of scrambled faces (SF), 15 s scrambled pictures (SP) and 15 s “*” for rest. Subjects were asked to name the drawings, and to count “1, 2”when shown the scrambled images, and to rest when “*” images were shown on the screen.

#### 2.3.3 Verbal fluency‐character

Verbal Fluency‐Character (VFC) contained five blocks, each block with two modules. Each module lasted 30 s. In the task module, a Chinese character would be shown on the screen, such as “天”(sky), and the participants were asked to make as many words containing “天” as they were able, e.g. “天空” (sky), “天气”(weather),”天文”(astronomy), etc. They rested when “*” images were shown.

#### 2.3.4 Verbal fluency‐letter

Verbal Fluency‐Letter (VFL) had five blocks, each block consisting of two modules, with 30 s per module. Stimulation tasks were Chinese consonant initials, requiring participants to name as many Chinese characters as possible, such as for the consonant initial “M”, Chinese characters could be “Ma”(妈, mother), “Mao”(毛, hair), “Mu”(幕, screen), etc. They rested when “*” images were shown.

Task (1), (3), (4) took 300 s each, and task (2) 375 s. There was a 10 s pre scan before each task. All tasks were designed using a professional task design program (Bonelli et al., 2011; Powell et al., 2006) and projected onto a screen at the foot of the MRI scanner couch.

The subjects viewed the projections through a mirror on the head coil, and heard instructions through an MRI compatible voice system.

### 2.4 Data analysis

Image processing and analysis data used SPM8 (RRID:SCR_007037; Wellcome Department of Cognitive Neurology, http://www.fil.ion.ucl.ac.uk/spm/) with the following key steps:

#### 2.4.1 Realign

In order to correct head movements, and to generate corrected and average images, we realigned all the functional images with the first phase functional images using a 3D sinc interpolation algorithm.

#### 2.4.2 Normalize

All images were normalized to our template (comprising 30 healthy volunteers, 15 patients with left hippocampal sclerosis and 15 patients with right hippocampal sclerosis, using the standard MNI brain as reference, and using nonrigid transformation to obtain the mean standardized functional parameters).

#### 2.4.3 Smooth

Normalized functional images were smoothed with a Gaussian function (full width at half maximum, FWHM = 6 mm).

We used the general linear model (GLM) to estimate the parameters in the time series of functional images. We restricted our analysis to temporal and frontal lobes and so activation levels were defined as *p *< .001. Group analyses were done using 2nd‐level analysis with single sample *t* test built‐in SPM8, calculating all of the data within group to distinguish activated and deactivated areas by a full factorial design. For individual subjects, we considered activations with *Z* > 2.5 and >10 voxels extent.

We recorded the Peak MNI coordinate (PMC), locations (maximum activated regions), sizes (Number of Voxels, NV), and peak intensity of language functional areas activated by different language stimulation tasks using Xjview (http://www.alivelearn.net/xjview8/).

### 2.5 Lateralization index

On the basis of individual and group analysis by SPM8, we calculated the lateralization index (LI) in frontal and temporal lobes by the software of LI (Wilke & Lidzba, 2007). We used a bootstrap approach, with four custom inclusive masks, an exclusive mask Midline (±5 mm), and others by bootstrap default settings. The four inclusive masks were constructed in house and comprised a frontal lobe mask (comprising inferior frontal gyrus and middle frontal gyrus), anterior and posterior temporal lobe masks, and a temporal lobe mask, which was a combination of anterior and posterior temporal lobe masks. Weighted mean values were obtained for each subject and group data.

To determine the correlation of LIs in the frontal and temporal lobe of each contrast, Pearson correlations were performed when all the variables were normally distributed, as verified by Kolmogorov–Smirnov Test. All statistical analyses were performed using SPSS16.0 (RRID: SCR_002865; SPSS Inc., Chicago, IL).

## 3 Results

### 3.1 Subjects

All the 19 healthy volunteers (11 female) were Chinese and had been living for an average 34.7 ± 34.0 months (range 1–120 months) in the UK. Their mean age was 29.6 ± 11.3 years (range 19–59 years). All were native Mandarin speakers. Five (26.3%) had high school education, 7 (36.8%) had an undergraduate degree, 7 (36.8%) had a postgraduate degree. According to the Edinburgh handedness questionnaire, 16 (84.2%) were right handed and 3 (16%) were ambidextrous. The average handedness score was 58.4 ± 30.9 (Table 1). No subjects had a history of neurological or psychiatric disturbance.

**Table 1:** Demographic data of the subjects

No.	Age (year)	Gender	Education level	English level	Months in UK	Handedness score^a^	Handedness
1	33	F	C	2	12	83	R
2	27	M	P	2	1	83	R
3	36	F	P	2	12	58	R
4	39	F	C	2	84	83	R
5	39	M	C	1	60	75	R
6	28	M	P	3	72	50	R
7	20	M	U	3	36	58	R
8	20	M	P	2	3	50	R
9	21	F	U	2	12	58	R
10	19	F	U	2	12	50	R
11	19	F	U	3	24	20	B
12	41	M	P	3	120	91	R
13	59	F	C	1	1	83	R
14	25	M	P	3	18	100	R
15	19	F	U	3	84	75	R
16	44	F	C	1	24	33	B
17	20	F	U	3	48	−8	B
18	18	F	U	3	24	−8	B
19	35	M	P	2	12	75	R

Gender F = female, M = male; Education Level: C<=College, U = Undergraduate, P = Postgraduate; English Level: 1 = IELTIS:<5, 2 = IELTIS:5‐7, 3 = IELTIS: >=7; Handedness: R = right handedness, L = left handedness, B = bilateral handedness.

a By the Edinburgh Hand Preference Inventory (Oldfield, 1971).

### 3.2 Group activations in temporal and frontal lobes

**Figure 1:**
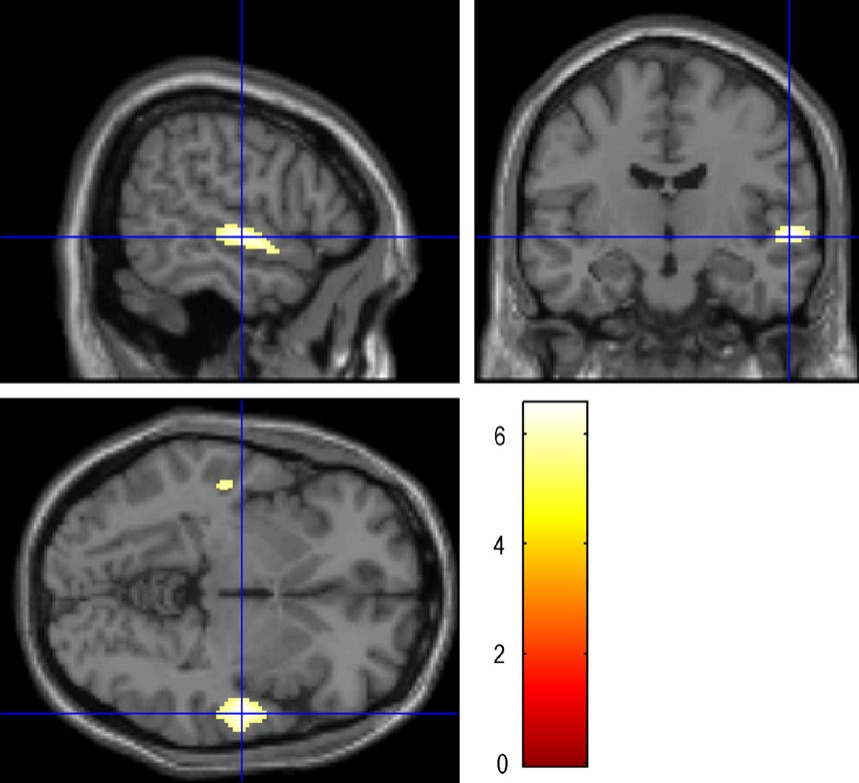
Auditory Naming: the maximum activation was located in the STG‐R (58 −12 −4), with the number of voxels 3157 and the peak intensity of 6.54. STG‐R: Right superior temporal gyrus

**Figure 2:**
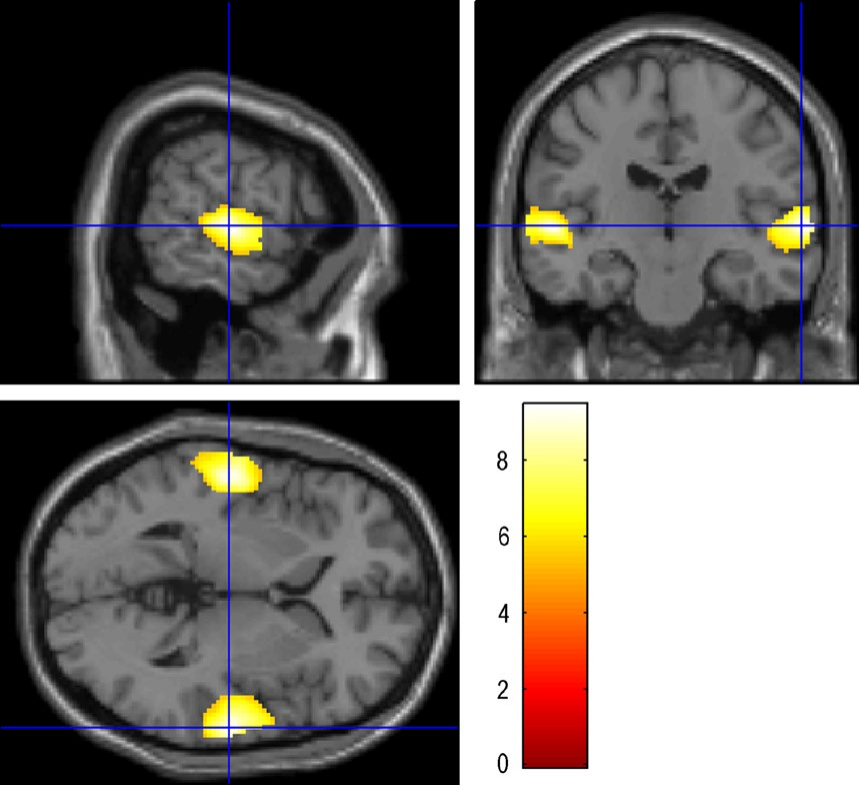
Auditory Naming – auditory reverse: the maximum activation was located in the STG‐R (64 −18 2), with the number of voxels 5721 and the peak intensity of 9.43. STG‐R: Right superior temporal gyrus

Auditory naming activated the right superior temporal gyrus (Fig. 1) and fusiform gyri, and the left middle temporal gyrus (Table 2). Auditory naming‐auditory reverse activated the left and right superior temporal gyrus (Fig. 2) and the right inferior temporal gyrus. For auditory naming and auditory naming‐auditory reverse, the main activation clusters were both located in right superior temporal gyrus.

**Table 2:** Group activations of all subjects

	NV	PMC	MAR	Peak intensity
AN	3157	58 −12 −4	STG‐R	6.54
	1682	−54 −20 0	MTG‐L	5.92
	33	40 −10 −30	FuG‐R	3.13
AN‐AR	5900	−56 −14 0	STG‐L	9.19
	5721	64 −18 2	STG‐R	9.43
	29	42 −8 −30	ITG‐R	2.89
PN	98	−40 10 24	IFG‐L	3.23
	375	58 −24 −2	STG‐R	4.38
	86	−58 −14 −4	MTG‐L	2.96
PN‐SP&SF	34	−40 10 24	IFG‐L	2.88
	34	56 −14 −6	STG‐R	2.97
	1165	−36 −46 −24	FuG‐L	4.49
VFC	2965	−10 66 6	SFG‐L	8.39
	966	34 28 50	MFG‐R	5.47
	808	−48 18 32	IFG‐L	5.43
	1045	−56 −12 −18	MTG‐L	5.95
VFL	223	22 −10 66	SFG‐R	5.93
	2659	−46 6 24	IFG ‐L	8.51
	26	46 22 14	IFG‐R	4.02
	47	−48 −28 16	STG‐L	3.98
	2464	−56 −8 −24	MTG‐L	9.09
	62	−24 −14 −28	PHG‐L	3.99

NV, Number of voxels; PMC, Peak MNI coordinate; MAR, Maximum activated region; AN, auditory naming; AR, auditory reversed; PN, picture naming; SP, scrambled pictures; SF, scrambled faces; VFC, verbal fluency‐Character; VFL, verbal fluency‐Letter.

Group analysis for all the 19 subjects of different language tasks, confined to temporal and frontal lobes, showing the activated region, number of activated voxels, peak MNI coordinate and peak intensity.

The strongest activations are highlighted in orange.

**Figure 3:**
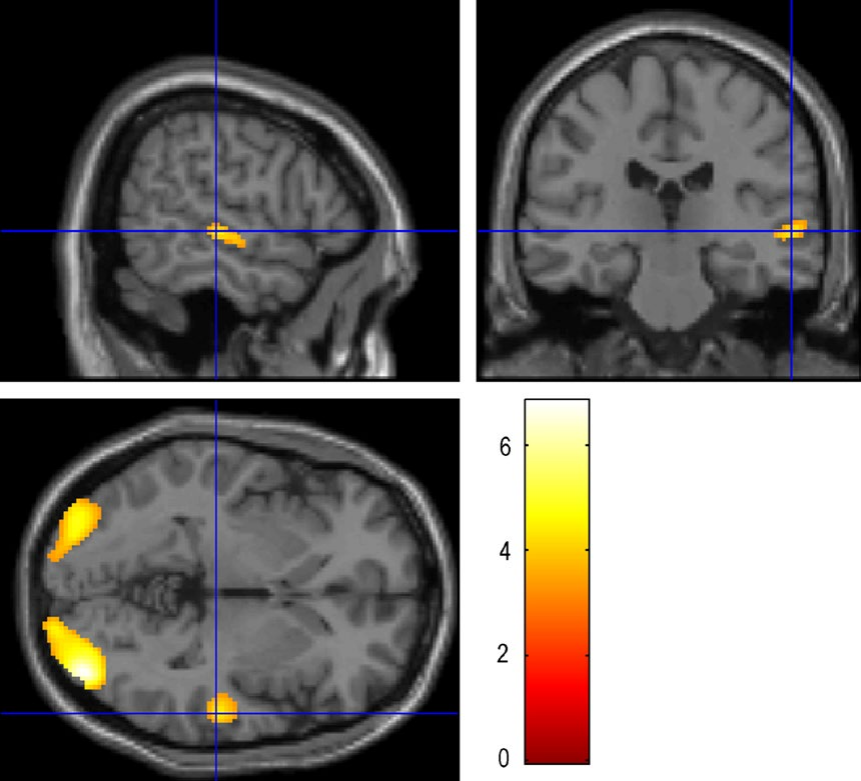
Picture Naming: the maximum activation was located in the STG‐R (58 −24 −2), with the number of voxels 375 and the peak intensity of 4.38. STG‐R: Right superior temporal gyrus

**Figure 4:**
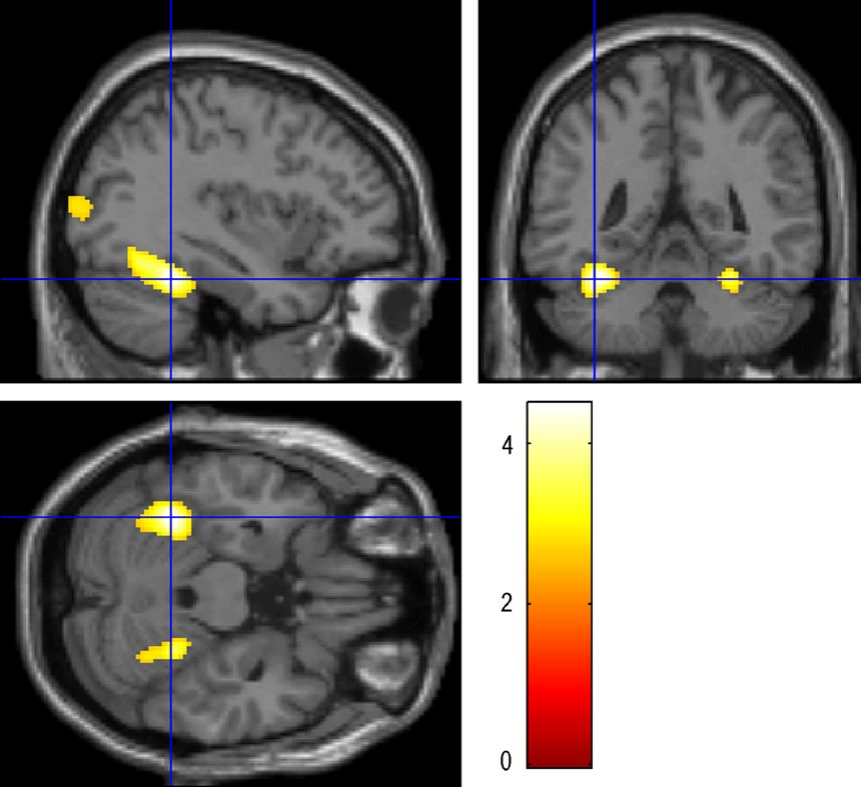
Picture naming‐scrambled pictures and faces:the maximum activation was located in the FuG‐L (−36 −46 −24), with the number of voxels 1165 and the peak intensity of 4.49. Fug L: Left fusiform gyrus

Picture naming activated the right superior temporal gyrus (Fig. 3) and the left middle temporal gyrus and inferior frontal gyrus. Picture naming‐scrambled pictures and faces activated the left fusiform gyrus and inferior frontal gyrus (Fig. 4), and the right superior temporal gyrus. For picture naming and picture naming‐scrambled pictures and faces, the activation clusters were more limited and weaker than those of deactivation clusters.

**Figure 5:**
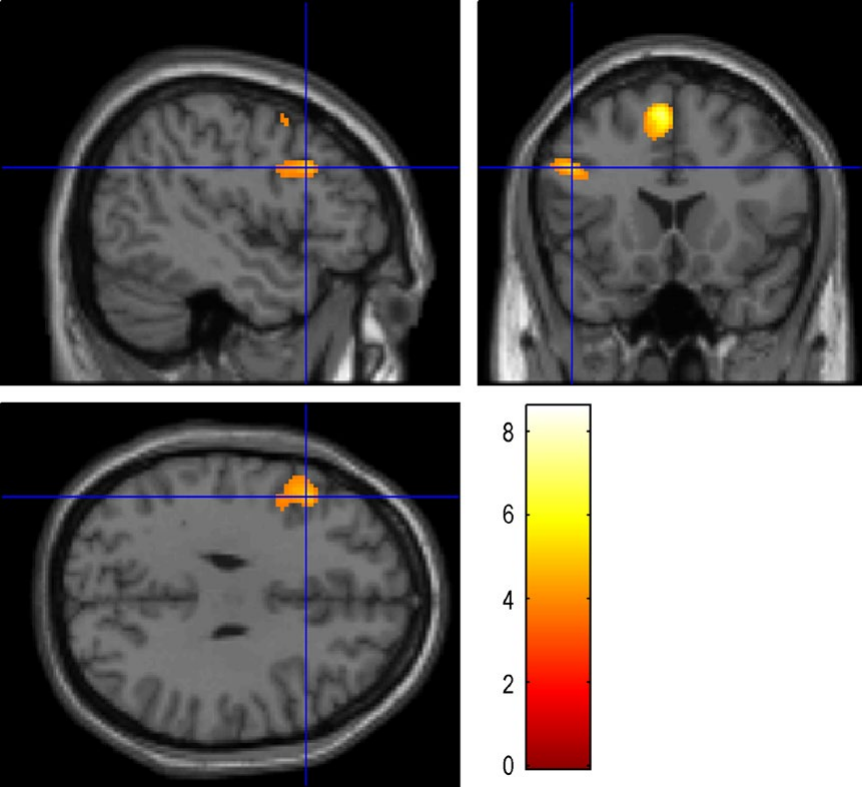
A group analysis of verbal fluency for characters shows that the activated region located at IFG‐L (−46 18 30) with the number of voxels of 808 and peak intensity of 5.43. FG‐L: Left frontal gyrus. Also activation in Left superior frontal gyrus

Verbal fluency for characters activated the left superior frontal gyrus, inferior frontal gyrus and middle temporal gyrus and the right middle frontal gyrus (Fig. 5).

Verbal fluency for letters activated the left inferior frontal gyrus, superior temporal gyrus, middle temporal gyrus and the parahippocampal gyrus, and the right superior frontal gyrus and inferior frontal gyrus.

For Verbal fluency for characters and Verbal fluency for letter tasks both had a more extensive and strong activation clusters than those of auditory naming and picture naming.

### 3.3 Group deactivations in temporal and frontal lobes

Auditory naming deactivated the left middle frontal gyrus and the inferior frontal gyrus and the right middle frontal gyrus (Table 3).

**Table 3:** Deactivations in all inferior subjects

	Deactive			
	NV	PMC	MAR	Peak intensity
AN	12	−38 44 4	IFG‐L	2.60
	991	−30 32 52	MFG‐L	5.57
	119	48 44 6	MFG‐R	4.46
AN‐AR	87	34 60 0	MFG‐R	3.40
	15	50 −2 −28	MTG‐R	2.76
	16	−46 −42 −26	ITG‐L	3.09
	16	22 0 −36	PHG‐R	2.89
PN	6798	−28 48 −8	MFG‐L	5.07
	449	34 12 62	MFG‐R	3.78
	12	−46 46 12	IFG‐L	2.64
	10	40 32 10	IFG‐R	2.63
	1512	−56 −32 −14	MTG‐L	6.05
PN‐SP&SF	27	20 64 8	SFG‐R	2.73
	275	−36 54 −2	MFG‐L	4.35
	1585	−50 −8 0	STG‐L	4.83
VFC	2155	−10 66 4	SFG ‐L	8.48
	748	38 22 50	MFG‐R	5.52
	822	−56 −12 −18	MTG‐L	5.92
	4775	50 −4 −28	MTG‐R	8.40
VFL	19	22 −10 66	SFG‐R	4.52
	18	−36 26 −24	STG‐L	4.74
	1224	−56 −8 −24	MTG‐L	6.92

NV, Number of voxels; PMC, Peak MNI coordinate; MAR, Maximum activated region; AN, auditory naming; AR, auditory reversed; PN, picture naming; SP, scrambled pictures; SF, scrambled faces; VFC, verbal fluency‐Character; VFL, verbal fluency‐Letter.

Deactivations in all 19 subjects, confined to temporal and frontal lobes, showing the deactivated region, number of voxels, peak MNI coordinate, and the peak intensity.

The locations of strongest deactivations are highlighted in orange.

Auditory naming‐auditory reverse deactivated the left inferior temporal gyrus and the right middle frontal gyrus, middle temporal gyrus and parahippocampal gyrus.

For auditory naming and auditory naming‐auditory reverse, the deactivation clusters were remote from the activation clusters, and weaker than those of activations.

Picture naming deactivated the left middle temporal gyrus, inferior frontal gyrus, middle frontal gyrus and the right inferior frontal gyrus and middle frontal gyrus, which were more extensive and strong than those of activations.

Picture naming‐scrambled pictures and faces deactivated the left superior temporal gyrus and middle frontal gyrus and the right superior frontal gyrus.

Verbal fluency for characters deactivated the left superior frontal gyrus and middle temporal gyrus and the right middle frontal gyrus and middle temporal gyrus.

Verbal fluency for letters deactivated the left middle temporal gyrus and superior temporal gyrus and the right superior frontal gyrus.

It's very interesting that, for verbal fluency for characters and verbal fluency for letters, the activation and deactivation were the same brain areas, left superior frontal gyrus, and left middle temporal gyrus, respectively.

**Figure 6:**
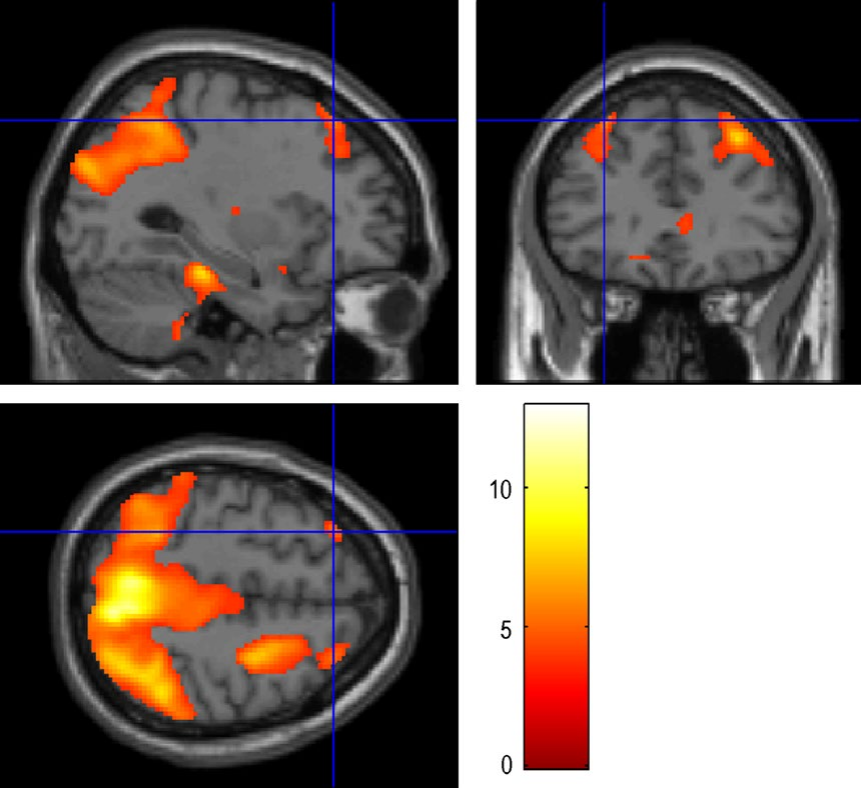
Auditory naming deactivations: the maximum deactivation was located in the MFG‐L (−30 32 52), with the number of voxels 991 and the peak intensity of 5.57

We restricted quantitative analysis to the temporal and frontal lobes. It was also evident that there was deactivation of parietal association areas (Fig. 6).

### 3.4 Individual subject results

There was some inconsistency of activations in individual subjects (Table 4). At a threshold of Z > 2.5, 10 voxels extent, activations were seen in >50% subjects for :

**Table 4:** Global maxima activation (GMA) of the individual subjects (N [%])

Contrast	AN	AN‐AR	PN	PN‐SP&SF	VFC	VFL
SFG‐L	2 (10.5)	8 (42.1)	6 (31.6)	4 (21.1)	3 (15.8)	3 (15.8)
SFG‐R	4 (21.1)	4 (21.1)	4 (21.1)	2 (10.5)	5 (26.3)	3 (15.8)
MFG‐L	6 (31.6)	3 (15.8)	6 (31.6)	6 (31.6)	9 (47.4)	8 (42.1)
MFG‐R	10 (52.6)	7 (36.8)	7 (36.8)	5 (26.3)	8 (42.1)	9 (47.4)
IFG‐L	7 (36.8)	6 (31.6)	6 (31.6)	10 (52.6)	6 (31.6)	3 (15.8)
IFG‐R	8 (42.1)	5 (26.3)	6 (31.6)	4 (21.1)	9 (47.4)	5 (26.3)
STG‐L	8 (42.1)	3 (15.8)	9 (47.4)	6 (31.6)	5 (26.3)	5 (26.3)
STG‐R	15 (78.9)	3 (15.8)	10 (52.6)	4 (21.1)	7 (36.8)	9 (47.4)
MTG‐L	11 (57.9)	7 (36.8)	6 (31.6)	8 (42.1)	8 (42.1)	7 (36.8)
MTG‐R	8 (42.1)	5 (26.3)	3 (15.8)	4 (21.1)	5 (26.3)	4 (21.1)
ITG‐L	5 (26.3)	2 (10.5)	4 (21.1)	9 (47.4)	1 (5.3)	5 (26.3)
ITG‐R	3 (15.8)	0 (0.0)	0 (0.0)	3 (15.8)	2 (10.5)	2 (10.5)
FuG‐L	0 (0.0)	1 (5.3)	4 (21.1)	2 (10.5)	0 (0.0)	0 (0.0)
FuG‐R	1 (5.3)	1 (5.3)	0 (0.0)	4 (21.1)	2 (10.5)	1 (5.3)
PHG‐L	1 (5.3)	1 (5.3)	1 (5.3)	1 (5.3)	1 (5.3)	0 (0.0)
PHG‐R	1 (5.3)	0 (0.0)	2 (10.5)	1 (5.3)	0 (0.0)	1 (5.3)
HC‐L	0 (0.0)	0 (0.0)	1 (5.3)	0 (0.0)	1 (5.3)	1 (5.3)
HC‐R	1 (5.3)	3 (15.8)	2 (10.5)	2 (10.5)	0 (0.0)	0 (0.0)

AN, auditory naming; AR, auditory reversed; PN, picture naming; SP, scrambled pictures; SF, scrambled faces; VFC, verbal fluency‐Character; VFL, verbal fluency‐Letter; SFG, superior frontal gyrus; MFG, middle frontal gyrus; IFG, inferior frontal gyrus; STG, superior temporal gyrus; MTG, middle temporal gyrus; ITG, inferior temporal gyrus; FuG, fusiform gyrus; PHG, parahippocampal gyrus; HC, hippocampus; R, right; L, left.

Main effects from the different language paradigms and their specified contrasts, in temporal and frontal regions, indicating the numbers and percentage of subjects with activations greater than a threshold of T > 2.5, with extent >10 voxels. Activations in >50% subjects are highlighted in orange.

Auditory naming, in the right superior temporal gyrus (78.9%), right middle frontal gyrus (52.6%), and the left middle temporal gyrus (57.9%).
Picture naming, in the right superior temporal gyrus (52.6%).
Picture naming – scrambled pictures and scrambled faces (PN‐SP and SF), in the left inferior frontal gyrus (52.6%).

### 3.5 Lateralization indices in temporal and frontal lobes

**Figure 7:**
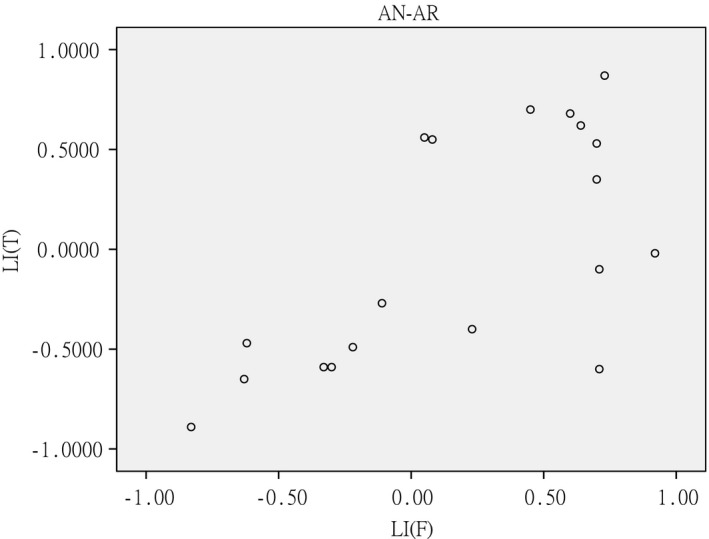
Scatter plot of Lateralization Index of AN‐AR in temporal and frontal lobe for all 19 subjects (*r *=* *.661, *p *=* *.002). LI(F):Lateralization index of frontal lobe, LI(T): Lateralization index of temporal lobe

In the group analysis, all the contrasts showed a left hemispheric dominance in frontal lobe. In the temporal lobe, the hemispheric dominance differed for different contrasts, (Table 5). For all the 19 individual subjects, there were a good correlation of dominance in frontal and temporal lobe of auditory naming and auditory naming‐auditory reverse (AN‐AR) (Table 6, Fig. 7).

**Table 5:** Lateralization index results for group analysis using frontal and temporal masks

	FL	TL	ATL	PTL
AN	0.84 ± 0.05	−0.42 ± 0.17	−0.29 ± 0.15	−0.35 ± 0.20
AN‐AR	0.24 ± 0.15	−0.12 ± 0.04	−0.04 ± 0.13	−0.01 ± 0.07
PN	0.96 ± 0.01	−0.10 ± 0.02	0.17 ± 0.08	−0.13 ± 0.05
PN‐PS&SF	0.95 ± 0.04	0.69 ± 0.19	0.37 ± 0.26	0.75 ± 0.20
VFC	0.96 ± 0.04	0.96 ± 0.01	0.95 ± 0.01	0.97 ± 0.01
VFL	0.98 ± 0.02	0.93 ± 0.04	0.90 ± 0.04	0.94 ± 0.03

FL, Frontal lobe (middle and inferior frontal gyri); TL, Temporal lobe (This is a combination of the two masks below); ATL, Anterior temporal lobe; PTL, Posterior temporal lobe.

**Table 6:** Correlation of LIs between the temporal and frontal lobes

	Normal distribution test		Pearson correlation	
	Z	*p*	R	*p*
AN	0.530	.941	0.589	.008
AN‐AR	0.853	.460	0.661	.002
PN	0.722	.675	0.172	.481
PN‐PS&SF	0.745	.635	0.218	.370
VFC	1.143	.147	0.240	.323
VFL	0.755	.619	0.441	.059

A *p* value of <.05 was considered statistically significant.

## 4 Discussion

### 4.1 Features of Chinese Language

Chinese characters are a symbol system, containing abundant cultural factors and reflecting cultural characteristics of ethnic Han. There are great differences between Chinese and English in pronunciation, intonation, grammar, vocabulary, and expressions. Chinese characters have no consonant cluster and are dominated by vowels, and there are tones for differing meaning and the main word‐formation method is word root of recombination (Ding, Liu, McBride, & Zhang, 2015; Liu, 2013; Xia & Andrews, 2014). Chinese characters lack morphological changes and an important feature of grammar for Chinese characters is the word order and functional word, and there is no simple corresponding relationship between word class and grammar. The combination of the words is subject to the semantic context. A Chinese language fMRI study is difficult to conduct, because of the above particularities and complexities.

### 4.2 Language paradigms of fMRI in Chinese

#### 4.2.1 Auditory naming

Auditory naming required subjects to name objects after hearing a description. This task was designed simply and the duration of stimulation reactions was long. It uses the levels of vocabulary that would be frequently used in daily life. Balsamo et al. (2002) discovered that the brain regions significantly activated in the process of listening comprehension, included the left superior, middle, inferior temporal, superior frontal, fusiform gyri and left lingual gyrus. Some reports found that auditory naming was more sensitive than picture naming in exploring naming difficulties caused by temporal lobe pathological damages. The naming speed and accuracy were reduced in 69% TLE patients via auditory naming, whereas picture naming speed and accuracy were decreased in only 38% and 40% TLE patients, respectively (Emerton, Gansler, Sandberg, & Jerram, 2014). The maximum activation with auditory naming in this study was located in the right superior temporal gyrus. Its peak intensity was about 1.5‐fold of picture naming task, and activation clusters of auditory naming had much more number of voxels than those of picture naming. Activations of auditory naming – auditory reverse, intended to negate the activations associated with hearing and subtle movements, were found bilaterally and symmetrically in the right and left superior temporal gyrus. The deactivation clusters were predominantly in the inferior and middle frontal gyrus of the frontal lobe, which was remote from the activation clusters.

#### 4.2.2 Picture naming

Picture naming is a basic element during language development. The earliest speech was formed from naming objects or human faces in childhood. Along with language development, initially formed object names were usually saved in the right cerebral hemisphere and gradually moved into the left cerebral hemisphere for further grammar and speech processing (Holland et al., 2001; Szaflarski et al., 2011). Prior studies showed that picture naming mainly activated lateral occipital lobe (BA18, 19), occipital pole (BA17), middle and inferior frontal lobe gyri (BA46, 47), and sometimes Broca's region (BA44, 45) (Chakraborty, Sumathi, Mehta, & Singh, 2012; Stippich et al., 2007; Yoon, Chung, Kim, Song, & Park, 2006). The occipital lobe (BA19) and temporal lobe (BA37) were activated when reading notional words (Tan et al., 2001). The picture naming task required subjects to consider each object presented, without too much semantic association and logical thinking processes. Therefore, semantic activations were relatively few, and picture naming only acted as a supplementary for other language tasks or was suitable for dysgnosia patients. The activated region on the picture naming task in this study was the same as above mentioned reports with maximum activated regions located in right superior temporal gyrus. Compared with another three language paradigms in this study, the activation clusters of picture naming were more limited, and the peak intensities were weaker. The picture naming‐scrambled pictures and faces contrast was intended to negate the effects of visual inputs and activated left inferior frontal gyrus and fusiform gyrus, and the right superior temporal gyrus.

#### 4.2.3 Verbal fluency

Verbal fluency was mainly used for evaluation of brain language execution and control processes. Neuropsychological studies revealed that verbal fluency mainly relied on the coordination between the frontal lobe and temporal lobe in the left hemisphere, and through different neural circuits to complete the letter or character naming fluency. When carrying out a test of naming fluency, the left middle frontal gyrus and fusiform gyrus were significantly activated (Birn et al., 2010). Fu et al. (2002) discovered that if the task difficulty was increased, the dorsal anterior cingulate cortex would be activated in healthy volunteers. The verbal fluency task mainly depended on the integrity of the language network function and activated the frontal lobe, including the dorsal lateral anterior cortex of frontal lobe. Kircher, Nagels, Kirner‐Veselinovic, & Krach (2011) found that the left inferior frontal gyrus, middle and superior temporal gyrus and the right cerebellum were all activated with language tasks such as verbal fluency, classification naming fluency and rhyme fluency. The activated areas of verbal fluency‐letter in this trial mainly include the left inferior frontal gyrus and the left middle temporal gyrus, whereas the activated areas of verbal fluency‐character mainly include the left superior frontal gyrus.

In this study, the two verbal fluency tasks, activation and deactivation clusters were very extensive and the peak intensities were very strong, suggesting that the two language paradigms were effective to activate the language functional areas in Mandarin speakers. We hypothesized that this could be attributed to Chinese characters, as a symbol and picture system, need more semantic segmentation. In our study, it is very interesting that the activation and deactivation were found in the same brain region for verbal fluency. In general, the deactivation, referring to a reduction in blood oxygenation‐level‐dependent signal during task performance relative to the baseline, could be detected in areas both close to or remote from the activated areas (Gusnard & Raichle, 2001). The deactivated middle temporal gyrus we observed was part of a ‘‘default network’’ (Raichle et al., 2001), this region was also activated in the task conditions. The present findings are consistent with the notion that parts of these regions are disengaged during the performance of cognitive tasks and that the magnitude of deactivation may increase in accordance with processing demands.

Different language tasks activate different language regions in the cerebral cortex, which will contribute to evaluation of the integrity of language function. This study has reduced the differences among voices and tones of Chinese language by combining several commonly used language stimulation tasks. It not only investigates the full coverage of language activated regions under different stimulation tasks but also objectively presents the specific locations, strength, and extent of maximum activated regions with different stimulation tasks. Therefore, the difference and significance of maximum activated regions under different stimulation tasks have been made clear, and our findings suggest these fMRI language paradigms have the potential to contribute to presurgical evaluations in native mandarin speakers and guide surgical decision making. Further research with larger numbers and with postoperative data will be needed to further explore their role further.

This research also has some shortcoming. This study has a relatively small sample and larger replication studies would be appropriate, as would test retest studies to determine the consistency of the results over time.

## Funding Information

This work was supported by the National Institute for Health Research (NIHR) University College London Hospitals Biomedical Research Centre (BRC).

## Conflicts of interest

All authors have no conflicts of interest in connection with the submitted article. They did not receive any outside funding or grants in support of your research for or preparation of the Work. The authors and the members of their immediate family did not received from any commercial entity any payments or any pecuniary, in kind, or other professional or personal benefits including stock, honoraria, or royalties or any commitment or agreement to provide such Benefits.
